# A comparison of functional outcomes following inpatient versus outpatient hip or knee arthroplasty

**DOI:** 10.1186/s13018-022-03270-7

**Published:** 2022-08-02

**Authors:** Larissa Sattler, Luke Kisaloff, Taiyler Cragnolini, Radd Peters, Wayne Hing

**Affiliations:** Bond Institute of Health and Sport, Bond University, 2 Promethean Way, Robina, QLD 4226 Australia

**Keywords:** Functional outcome measures, Knee Arthroplasty, Hip Arthroplasty, Outpatient Arthroplasty, Same-day discharge

## Abstract

**Background:**

The length of hospital stay after lower limb arthroplasty has rapidly decreased in the last decade, largely in part due to the rise of improved perioperative protocols, but also as a response to the increased economic demand associated with the rapid growth in hip and knee arthroplasty procedures. In line with this, the development of a new pathway after lower limb arthroplasty that allows for the surgery to be performed in an outpatient setting and permits for same-day discharge after the procedure is increasingly being offered. Although costs and complications between the inpatient and outpatient models have been compared, there appears to be little known about the effects on a patient’s physical function after undergoing hip or knee outpatient arthroplasty. Therefore, this systematic review aims to explore the available evidence for the effect on functional outcomes following inpatient versus outpatient hip or knee arthroplasty.

**Methods:**

This systematic review adhered to the PRISMA guidelines and was prospectively registered (https://osf.io/8bfae/). An electronic search of three online databases (PubMed, CINAHL and EMBASE) was conducted to identify eligible studies. All studies investigating inpatient and outpatient comparator groups, for a population of patients undergoing hip or knee arthroplasty, that assessed one or more functional outcomes, were included. A methodological quality appraisal was undertaken for the final studies contained in this review. A narrative synthesis of results is described along with quantitative outcomes presented in tables and figures.

**Results:**

A total of seven studies containing 1,876 participants were included in this review. Four studies assessed a THA population, two assessed TKA and one assessed both. Functional outcomes varied, with 20 different functional outcomes utilised, of which 18 were patient-reported tools. Results of functional outcomes offered mixed support for both inpatient and outpatient pathways.

**Conclusions:**

The results of this review suggest that outpatient or inpatient pathway selection for hip or knee arthroplasty should not be based on the superiority of functional outcomes alone. However, given there is growing evidence in support of an outpatient pathway in select patients with respect to cost savings and without any increase in complications, it could be proposed that an equivalency of post-operative function between the two settings makes same-day discharge favourable.

Publicly registered with Open Science Framework (https://osf.io/8bfae/).

**Supplementary Information:**

The online version contains supplementary material available at 10.1186/s13018-022-03270-7.

## Background

The prevalence of hip and knee osteoarthritis is climbing in line with increases in global lifespan and higher levels of obesity [1–3]. A rise in youth sporting injuries is also responsible for increased rates of posttraumatic osteoarthritis in younger adults [4, 5]. With osteoarthritis being the most commonly reported reason for undergoing lower limb arthroplasty it is not surprising that a subsequent surge in hip and knee arthroplasty procedures is predicted [6–10]. As the economic burden associated with the increase in these procedures grows, optimisation of health care resources and the development of sustainable perioperative delivery models are of critical consideration [6, 11–13].

One strategy for improving cost containment is by reducing the length of hospital stay after a hip or knee arthroplasty procedure. The financial incentive along with surgical advances and rapid recovery protocols has led to the number of average days a patient stays after arthroplasty surgery decline [10, 14–16]. In line with this trend for reducing the length of stay, an outpatient or same-day discharge surgery service, that can be managed in a hospital or ambulatory surgery facility, is being offered to select lower limb arthroplasty patients with increasing frequency [17–22].

Existing research suggests that along with decreased costs, there is also no increased risk of complications associated with same-day discharge after lower limb arthroplasty in appropriately selected patients [23–28]. For these reasons, utilisation of hip and knee arthroplasty in an outpatient setting has increased and is predicted to continue to do so [29–31]. However, typically there are select patient criteria that need to be met before undergoing lower limb arthroplasty in an outpatient setting. The recommended eligibility for outpatient surgery tends to include younger, more active patients with a lower number of comorbidities and who have social support on discharge, as such, an outpatient pathway may not be appropriate for individuals who do not meet these criteria [10, 17, 24, 30, 32]. Patients requiring an inpatient stay are more likely to require access to hygiene assistance, are at a greater risk for falls or need closer monitoring due to an increased risk of post-operative complications [33–35]. A risk assessment tool to help predict which patients may safely undergo same-day discharge has been developed, The Outpatient Arthroplasty Risk Assessment (OARA) score stratifies patients by nine medical categories to generate a risk category [36].

To date, most studies have focused on the comparison between inpatient and outpatient settings assessing the safety and success of the surgery as defined by costs and feasibility, or complications and readmissions [25, 37–41] Research investigating outcomes of function and physical performance appears to be far more limited. A recent review reported the effects on patient-reported outcome measures (PROMs) for same-day discharge patients following hip arthroplasty, however, only one included study had an inpatient comparator group [42]. Those undergoing arthroplasty in an outpatient setting have the potential to lack access to the included resources of those with a longer length of hospital stay, such as physical therapy and rehabilitative services or nursing care. Outpatient arthroplasty also relies on extensive preoperative patient education and advanced perioperative protocols for the success of the procedure. It could be suggested then that there is potential for a greater likelihood of decreased physical or self-reported functional outcomes in an outpatient population when compared to an inpatient group.

As rates of outpatient lower limb arthroplasty increase, research comparing the effects on functional outcomes to a traditional inpatient pathway could provide health care stakeholders and prospective patients with greater insight into the risks and benefits of each. If patient functional outcomes are equivalent or superior to those associated with an inpatient stay, then this combined with existing evidence to suggest the outpatient setting is both cost-effective and safe, would add further support for the uptake of the outpatient model in selected patients. Therefore, this systematic review aims to explore the available evidence for the effect on functional outcomes following inpatient versus outpatient hip or knee arthroplasty.

## Methods

This systematic review has been conducted in accordance with the Preferred Reporting Items of Systematic Reviews and Meta-Analyses (PRISMA) guidelines (Additional file 1) and the review protocol has been prospectively and publicly registered with Open Science Framework (https://osf.io/8bfae/) [43, 44].

### Study eligibility

Inclusion criteria were defined based on Population, Intervention, Comparison, and Outcome (PICO) method.

1. Population: Adults ≥ 18 years of age who have undergone joint arthroplasty, including Total Knee Arthroplasty, Unicompartmental Knee Arthroplasty, or Total Hip Arthroplasty of any surgical approach.
2. Type of Intervention: Arthroplasty surgery is performed as an outpatient procedure, either in an ambulatory surgery centre or hospital, as long as the patient discharges on the day of surgery.
3. Type of Comparison: Arthroplasty surgery performed in a hospital that includes an overnight stay as an inpatient.
4. Outcomes: At least one functional outcome was required, either a measure of physical performance or a patient-reported functional outcome measure could be utilised.

Exclusion criteria: (1) Articles not available in the English language and (2) Articles only available as an abstract or conference proceeding.

### Search strategy

Three databases were searched up to November 4th, 2021 (PubMed, CINAHL, EMBASE). Using the advanced search strategy acquired on PubMed, the Polyglot Search Translator was then used to convert these search terms into the polyglot strings necessary for CINAHL and EMBASE [45]. Keywords used for our search included inpatient, outpatient hip arthroplasty, knee arthroplasty and uni-compartmental knee arthroplasty. Associated synonyms were also acquired using mesh terms. The full search strategies utilised across the three databases are reported in full in Appendix Table 9.

### Study selection

Using the predetermined eligibility criteria, an initial search of titles and abstracts was conducted. Articles were imported to the Endnote referencing software and the Systematic Review Accelerator tool, Screenatron [46]. Prior to title and abstract screening, all duplicated studies were removed using the De-duplicator tool [47]. The screening of titles and abstracts was divided evenly and completed by three researchers (RP, LK, TC). Following this process, screening of full-text articles was performed independently by two researchers (RP, LK) who were blinded to each other’s decisions, to acquire the final studies included in this review. Any disagreement on final study inclusion was resolved by discussion with a third researcher (TC).

### Data extraction

An individual researcher (TC) initially extracted the data, with two researchers (RP, LK) reviewing it upon completion to minimise the chance of error. Information extracted from studies was recorded and saved in separate tables adapted from the Cochrane Collaboration Data Collection Form, identified from the Cochrane Handbook for Systematic Reviews of interventions [48]. The data collected included items that related to study population characteristics, outcomes assessed, and results of functional outcomes measures.

### Methodological quality assessment

A methodological quality appraisal was conducted on included articles in this systematic review, in line with recommended frameworks for conducting systematic reviews [43]. For assessing the quality of the individual articles included, the Joanna Biggs Institute (JBI) critical appraisal tools were utilised [49]. Two researchers (RP, TC) independently assessed the included articles using the relevant JBI appraisal tool that related to the study design and an agreement score was reported. Any disagreement on appraisal scores was resolved by discussion and consensus agreement with a third researcher (LK). The agreement score was converted into a percentage obtaining a quality grade; over 61% were considered of good methodological quality, between 45.4 and 61.0% were considered “fair” and < 45.4% were considered “poor” quality [50]. The Kappa coefficient statistic was used to measure the interrater reliability and agreement between the two researchers (RP, TC). Kappa coefficient agreement values range from near perfect, 0.81–1.00, substantial, 0.61–0.80, moderate, 0.41–0.60, fair, 0.21–0.40, and slight 0.0–0.2 [51].

### Results synthesis

Results have been presented for each study and grouped according to outcome measures. A narrative synthesis of the results of all studies is described along with the quantitative outcomes presented in Tables 4, 5, 6, 7 and 8. To aid comparison between studies, where effect measures are reported in an included study, means and standard deviations are recounted in the respective table for each functional outcome, along with the significance level (P value) between the inpatient and outpatient group results.

## Results

### Literature search and study characteristics

**Fig. 1:**
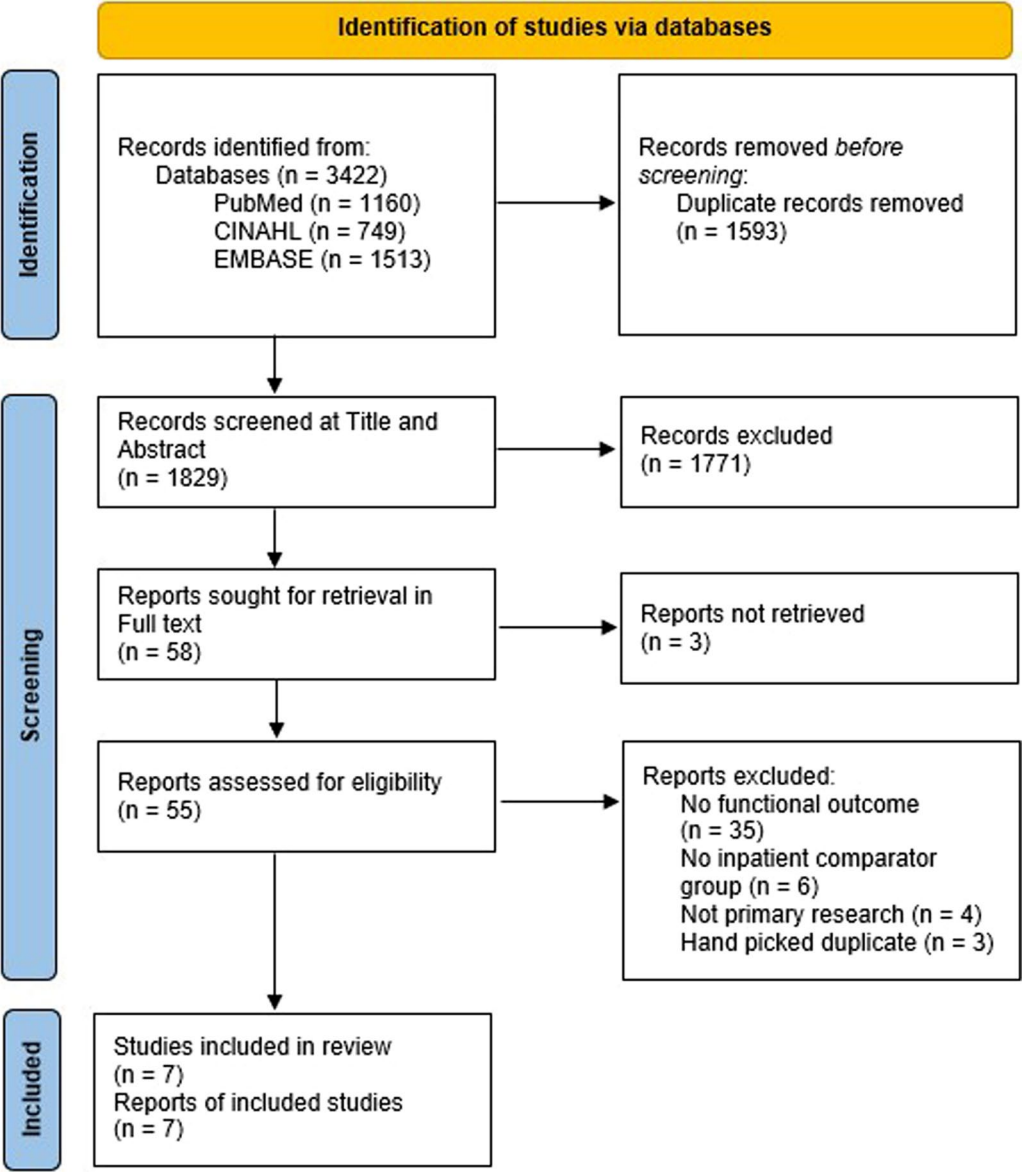
PRISMA flow diagram of study screening and selection

The initial search of the three databases resulted in a combined total of 3422 articles. Of those articles, 1,593 were removed as duplicates leaving 1829 to be reviewed at title and abstract level for eligibility. Following a second full-text screening of 55 articles, a final seven studies were included in this review [52–58]. The complete search and screening process is outlined in Fig. 1.

### Methodological quality assessment results

The articles included in this systematic review underwent a methodological quality appraisal, which was conducted independently by two researchers (RP, TC) and found all studies to be of good methodological quality (> 61%). The agreement between individual scores produced a Cohen’s Kappa result of 0.687, which demonstrated substantial agreement between the two authors (Appendix Table 10). Disagreements were resolved by discussion and consensus with a third researcher (LK) which produced a final Kappa score of 1.00. The full methodological critical appraisal results are reported in Appendix Table 10.

### Characteristics of included studies

Of the included studies, four assessed THA, two assessed TKA and one assessed both, however, no included study assessed UKA as part of their population. Study design varied, five provided a cohort study, one a randomised control trial and one a case–control study. A total of 1,876 participants were assessed, with the inpatient group representing a larger sample with 1043 participants. Overall, most participants were aged > 53 years, with a younger age range shown in the outpatient population. Female sex prevalence was greater in the inpatient population (55%) versus the outpatient population (43%). Study characteristics are reported in full in Table 1. Inclusion and exclusion criteria denoting patient eligibility for the outpatient pathway differed between studies, similarities within the criteria pertained to overall patient health relating to co-morbidities that would exclude the patient from an outpatient pathway. However, there was variation on specific eligibility criteria, such as age, with only two studies reporting on inclusion for this [54, 58]. The complete inclusion and exclusion criteria for all studies are reported in Table 2.

**Table 1:** Study overview and participant characteristics

Author, Year, Country	Title	Study Design	Level of Evidence*	Arthro-plasty	Participant numbers		Sex (M/F)		Age (Years)					
									OP			IP		
					OP	IP	OP	IP	Mean	Range	SD	Mean	Range	SD
Gabor, 2020, USA	Similar Outcomes After Hospital-Based Same-Day Discharge vs Inpatient Total Hip Arthroplasty	Retrospective Cohort	Level III-2	THA	415	548	202/213	196/352		≤ 59–91			≤ 59–91	
Goyal, 2016, USA	A Multicentre, Randomized Study of Outpatient versus Inpatient Total Hip Arthroplasty	Randomised Control Trial	Level II	THA	112	108	59/53	58/50	59.8	27–74	± 8.5	60.2	34–74	± 8.9
Gauthier-Kwan, 2018, Canada	Quality of Recovery, Post-discharge Hospital Utilization, and 2-YearFunctional Outcomes After an Outpatient Total Knee Arthroplasty Program	Prospective Cohort	Level III-2	TKA	43	43	29/14	22/21	62.5	50–75		62.5	51–74	
Husted, 2020, Denmark	Are functional outcomes and early pain affected by discharge on the day of surgery following total hip and knee arthroplasty?	Prospective Cohort	Level III-2	THA/TKA	92	169	53/39	80/89	60		± 11	62		± 11
Kolisek, 2009, USA	Comparison of Outpatient versus Inpatient Total Knee Arthroplasty	Prospective Cohort	Level III-2	TKA	64	64	40/24	40/24	55	42–64		55	42–64	
Rosinsky, 2020, USA	Outpatient vs. inpatient hip arthroplasty: a matched case–control study on a 90-day complication rate and 2-year patient-reported outcomes	Case–Control	Level III-2	THA	91	91	53/38	49/42	53.3	29–65	± 7.2	55.3	36–75	± 6.9
Zomar, 2021, England	Perioperative gait analysis after total hip arthroplasty: Does outpatient surgery compromise patient outcomes?	Prospective Cohort	Level III-2	THA	16	20	9/7	12/8	56.8		± 7.9	64.6		± 9.0

*ADL*—activities of daily living, *ASA*—American Society of Anaesthesiologists Physical Status Classification system, *BMI*—body mass index, *DOS*—day of surgery, *F*—Female, *M*—Male, *IP*—Inpatient, *OP*—Outpatient, *OA*—osteoarthritis, *SDD*—same day discharge, *nSDD*—Not Same-day discharge, *SD*—Standard Deviation, *THA*—total hip arthroplasty, *TKA*—total knee arthroplasty

*Level of evidence is based on the NHMRC evidence hierarchy

**Table 2:** Eligibility criteria for outpatient arthroplasty

Author, year	Inclusion Criteria	Exclusion Criteria
Gabor, 2020	Not currently on chronic anticoagulation	Assigned to SDD protocol but were not SDD (discharged before midnight on the day of surgery)
	No active coronary artery disease or active arrhythmias	Those who completed only one or neither of the preoperative or 12-week postoperative outcome surveys
	No active liver disease	
	No moderate or severe obstructive sleep apnoea, blood haemoglobin (greater/equal to)12 g/dL, BMI ≤ 40 kg/m^2^	
	Can ambulate independently	
	Patients willing to undergo pre-operative education and had support organised for discharge criteria	
Goyal, 2016	Primary THA without acute hip fracture or prior hardware that would be removed at the time of surgery	Revision THA or THA
	Unilateral THA	Preoperative BMI ≥ 40 kg/m^2^
	Preoperative BMI < 40 kg/m^2^	Age ≥ 75 years at the time of surgery
	Age < 75 years	Preoperative haemoglobin ≤ 10 g/dL if preoperative haemoglobin data were available
	Preoperative Haemoglobin > 10 g/dL	History of cardiopulmonary disease requiring acute inpatient monitoring
		Preoperative ambulatory status requires the use of a walker or wheelchair
		Chronic preoperative opioid medication uses or opioid addiction
		Limited or no assistance available at home after discharge from the hospital
		Any other condition or circumstance that would preclude rapid discharge from the hospital
Gauthier-Kwan, 2018	Patients undergoing primary TKA for end-stage osteoarthritis of the knee	Not Reported
	ASA score ≤ 3 with a stable medical profile	
	BMI < 45 kg/m^2^	
Husted, 2020	Does not suffer from sleep apnoea and had an ASA score of < 3	Patients not fulfilling discharge on the DOS
	Patients who were operated on as 1st or 2nd in the surgical theatre	
Kolisek, 2009	Discharge within 23 h of the procedure	History of diabetes, myocardial infarction, stroke, congestive heart failure, venous thromboembolism, cardiac arrhythmia, respiratory failure, or chronic pain requiring regular opioid medications
Rosinsky, 2020	Patient decision for outpatient preference	Not reported
	Hip OA which impaired ADLs and was refractory for minimum 3 months of conservative treatment	
	No significant comorbidities	
Zomar, 2021	18–75 years	BMI > 40
	Undergoing unilateral primary THA via direct approach	Unable to ambulate 10 m without gait aid before surgery
	Single surgeon	Undergone ipsilateral TKA
	Requiring surgery for the treatment of OA	Comorbidities of the lower extremities affecting gait

*ADL*—activities of daily living, *ASA*—American Society of Anaesthesiologists Physical Status Classification system, *BMI*—body mass index, *DOS*—day of surgery, *SDD*—same day discharge, *THA*—total hip arthroplasty, *TKA*—total knee arthroplasty, *OA*—osteoarthritis

### Outcomes assessed

A total of 20 functional outcome measures were identified across all studies (Table 3). The majority of these being PROMs with the visual analogue scale (VAS), Harris hip score (HHS) and patient-reported satisfaction score all presenting across three or more studies. The remaining outcomes were present across less than three studies, while only one study assessed measures of physical performance; gait analysis and timed up and go (TUG) [58].

**Table 3:** Frequency of functional outcome measures utilised across all studies

Functional outcome measure	Rosinsky, 2020	Gabor, 2020	Gauthier-Kwan, 2018	Husted, 2020	Kolisek, 2009	Goyal, 2016	Zomar, 2021	Total
*Patient Reported*								
Harris Hip Score (HHS)	✓					✓	✓	3
Patient Satisfaction Score	✓			✓	✓			3
Veterans Rand-12 Item Health Survey (VR-12P)	✓	✓						2
Veterans Rand-12 Item Health Survey (VR-12 M)	✓	✓						2
Short form: Physical (SF-12P)	✓						✓	2
Short form: Mental (SF 12 M)	✓						✓	2
Western Ontario and McMaster Universities Osteoarthritis Index (WOMAC, 4-scale)			✓				✓	2
Modified Harris Hip Score (mHHS)	✓							1
Forgotten Joint Score (FJS)	✓							1
Hip Disability and Osteoarthritis Outcome Score for Joint Replacement (HOOS JR)		✓						1
Quality of Recovery Scores (QOR-9)			✓					1
Knee Injury and Osteoarthritis Outcome Score (KOOS, 5-subscale)			✓					1
Oxford Knee Score (OKS)				✓				1
Oxford Hip Score (OHS)				✓				1
Knee Society Score (KSS, 2-subscale)					✓			1
Knee Range of Motion (KROM)					✓			1
*Pain intensity*								
Visual Analog Scale (VAS)	✓	✓		✓		✓	✓	5
Numeric Pain Rating Scale (NRS)			✓	✓				2
*Physical Function*								
Timed up & go (TUG)							✓	1
GAIT rite							✓	1
✓ outcome measure included								

### Summary of evidence

The combined results of all studies are reported in Tables 4, 5, 6, and 7 (Table 8).

**Table 4:** Results of functional outcome measures across three or more studies

Author	Arthroplasty type	Timepoint	Mean (SD where reported)		Statistical significance (*P* value)
			Outpatient	Inpatient	
*Visual Analog Scale (pain intensity)*					
Gabor, 2020	THA	12 weeks—baseline average	− 4.8 (2.2)	− 5.1 (2.3)^†^	0.05*
Goyal, 2016	THA	Day of surgery	2.8 (2.5)	3.3 (2.3)	0.12
		Day after Surgery	3.7 (2.3)	2.8 (2.1)^†^	0.05*
		4 weeks	1.7 (1.9)	1.7 (1.9)	0.77
Husted, 2020	THA	POD1-7	NR	NR	> 0.05
	TKA rest	POD1	4.6	4.0	0.30
		POD2	5.2	3.6^†^	0.02*
		POD7	3.6	3.3	0.70
	TKA active	POD1	5.4	5.6	0.70
		POD7	4.6	4.7	0.90
Rosinsky, 2020	THA	2 years	1.0 (2.0)^†^	1.5 (2.2)	0.04*
Zomar, 2021	THA	Discharge	3.1 (0.5)^†^	4.6 (0.5)	0.04*
		2 weeks	2.6 (0.5)	2.5 (0.5)	> 0.05
		6 weeks	1.6 (0.5)	1.2 (0.5)	> 0.05
		12 weeks	1.0 (0.4)	0.6 (0.4)	> 0.05
*Harris Hip Score (Hip function)*					
Goyal, 2016	THA	4 weeks	75.0 (18)	75.0 (14)	0.77
Rosinsky, 2020	THA	2 years	92.3 (13.4)^†^	87.4 (15.6)	0.02*
Zomar, 2021	THA	12 weeks	96.3 (1.3)	95.8 (1.2)	> 0.05
*Patient Satisfaction Score*					
Husted, 2020	THA/TKA	4 weeks	75.0 (18)	75.0 (14)	0.77
Kolisek, 2009	TKA	2 years	92.3 (13.4)^†^	87.4 (15.6)	0.02*
Rosinsky, 2020	THA	12 weeks	96.3 (1.3)	95.8 (1.2)	> 0.05

*NR*—not reported, *HHS*—Harris Hip Score, *POD*—postoperative day, *SDD*—same day discharge, *nSDD*—not same day discharge, *SD*—standard deviation, *THA*—total hip arthroplasty, *TKA*—total knee arthroplasty, *VAS*—Visual Analog Scale

*Statistical significance =  < .05

^†^Result favouring group

**Table 5:** Results of functional outcome measures across less than three studies

Author	Time point	Outpatient	Inpatient	Statistical significance (*P* value)
*Numeric Pain Rating Scale (mean/SD)*				
Gauthier-Kwan, 2018	POD 1	3.6 (1.5)	3.2 (2.2)	0.20
	POD 7	2.9 (1.9)	3.0 (1.9)	0.82
	POD 14	2.9 (2.1)	2.9 (1.9)	0.86
	POD 21	2.3 (1.8)	2.4 (1.8)	0.80
	POD 28	2.1 (1.7)	2.0 (1.9)	0.60
Husted, 2020	NR	> 0.05		
*Forgotten Joint Score Mean (Mean/SD)*				
Rosinsky, 2020	2 years	80.0 (22.7)	71.2 (30.8)	0.16
*Modified Harris Hip Score; Mean (SD)*				
Rosinsky, 2020	2 years	91.5 (14.7)^†^	86.2 (17.1)	0.02*
*Hip Disability and Osteoarthritis Outcome Score for Joint Replacement (Mean/SD)*				
Gabor, 2020	12 weeks—baseline Average	29.9 (16.7)	31.3 (16.7)	0.29
*Knee Range of Motion (Mean Degrees)*				
Kolisek, 2009	24 months	123	121	0.28
*Oxford Knee Score (Mean)*				
Husted, 2020	3 months	32	31	0.60
	1 year	39	38	0.50
*Oxford Hip Score (Mean)*				
Husted, 2020	3 months	39	37	0.10
	1 year	43	43	1.00
*Quality of Recovery Scores (Mean/SD)*				
Gauthier-Kwan, 2018	POD 1	15.4 (2.0)^†^	13.9 (2.8)	0.01*
	POD 3	16.4 (1.8)	15.6 (2.5)	0.30
	POD 7	16.4 (1.8)	16.2 (2.1)	0.62
	POD 14	15.8 (2.2)	16.4 (2.0)	0.12
	POD 21	16.2 (3.1)	16.6 (2.0)	0.59
	POD 28	16.4 (3.0)	16.8 (1.8)	0.44

*NR*—not reported, *POD*—post operative day, *SD*—standard deviation

*Statistical significance =  < .05

^†^Result favouring group

**Table 6:** Results of outcome measures with subscales

Author, year	Time point	Subscale	Outpatient	Inpatient	Statistical significance (*P* value)
*Knee and Injury Osteoarthritis Outcome Score Mean (SD)*					
Gauthier-Kwan, 2018	1 year	Symptoms	73.1 (15.5)	79.2 (17.3)	0.11
		Pain	82.1 (16.2)	83.8 (18.0)	0.59
		ADL	86.2 (13.9)	85.4 (17.6)	0.81
		SR	60.2 (25.8)	54.7 (27.2)	0.53
		QoL	57.4 (25.9)	70.9 (23.2)	0.05
	2 year	Symptoms	80.2 (12.3)	79.6 (17.6)	0.56
		ADL	88.1 (13.4)	88.7 (14.8)	0.50
		Pain	89.5 (13.2)	88.1 (16.5)	0.96
		SR	61.5 (25.7)	64.0 (23.1)	0.77
		QoL	69.4 (19.3)	76.0 (24.2)	0.09
*Western Ontario and McMaster Universities Osteoarthritis Index Mean (SD)*					
Gauthier-Kwan, 2018	1 year	Pain	88.1 (13.0)	87.1(17.3)	0.78
		Stiffness	75.0 (13.7)	80.7 (18.5)	0.13
		Function	86.2 (13.9)	85.5 (17.6)	0.81
		Total	84.6 (12.0)	85.1 (16.8)	0.43
	2 year	Pain	92.6 (12.0)	87.7 (21.3)	0.31
		Stiffness	82.3 (16.4)	82.5 (20.0)	0.65
		Function	89.5 (13.2)	88.2 (16.5)	0.95
		Total	89.3 (12.5)	87.9 (15.9)	0.93
*Knee Society Score Mean*					
Kolisek, 2009	24 months	Knee Score	94	93	0.26
		Function Score	86	86	0.96

*ADL*—activities of daily living, *QoL*—quality of life, *SR*—sport and recreation, *SD*—standard deviation

Statistical significance =  < .05

**Table 7:** Results of Veteran Rand-12 and Short form-12

Author, year	Time point	Outpatient	Inpatient	Statistical significance (*P* value)	Outpatient	Inpatient	Statistical significance (*P* value)
		VR-12P mean (SD)			VR-12M mean (SD)		
Rosinsky, 2020	2 years	51.4 (8.9)	48.9 (10.6)	0.121	62.1 (5.5)	60.4 (8.0)	0.15
Gabor, 2020	12 weeks—baseline Average	4.3 (10.2)	6.1 (11.1)	0.040	14.1 (10.1)	14.4 (8.8)	0.65
		SF-12P mean (SD)			SF-12M mean (SD)		
Rosinsky, 2020	2 years	49.8 (9.5)	47.4 (11.0)	0.132	57.7 (5.4)	56.3 (7.9)	0.37
Zomar, 2021	2 weeks	31.5 (2.0)	31.1 (1.8)	> 0.05	56.8 (2.6)	50.9 (2.3)	> 0.05
	6 weeks	40.1 (2.7)	42.3 (2.4)	> 0.05	54.5 (2.2)	54.1 (2.0)	> 0.05
	12 weeks	45.6 (2.5)	45.3 (2.3)	> 0.05	56.9(1.8)	55.9 (1.6)	> 0.05

*SD*—standard deviation, *VR-12*—veterans rand-12, *SF-12*—short form-12

**Table 8:** Results of gait analysis including timed up & go

Author, year	Time point	Characteristic	Outpatient	Inpatient	Statistical significance
*Gait analysis mean (SD)*					
Zomar, 2021	12 weeks	Velocity (cm/s)	116.8 (3.9)	114.9 (3.4)	> 0.05
		Stride length (cm)	130.5 (2.9)	129.3 (2.6)	> 0.05
		Double limb support (% gait cycle)	27.6 (0.7)	28.8 (0.7)	> 0.05
		Single limb support (% of git cycle) operated limb	36.6 (0.5)	35.5 (0.5)	> 0.05
		Step length (cm) operated limb	66.7 (1.3)	65.3 (1.2)	> 0.05
		TUG (s)	8.54 (0.47)	9.35 (0.42)	> 0.05

*SD*—standard deviation, *TUG*—timed up & go

#### Pain intensity and function

Concerning decreased pain for the studies investigating a THA population, VAS scores between studies displayed results that were conflicting, with two studies favouring the inpatient group [52, 54] while two studies favoured the outpatient group[57, 58]. Timepoints of these assessments also varied, the results favouring the inpatient group were taken at time points less than three months after surgery, whereas the results favouring the outpatient group were assessed on the day of discharge and at a two-year follow-up. Only one study that included a TKA population reported significantly less pain between the two groups, which was on post-operative day two, and this favoured the inpatient group [55]. Rosinsky, 2020 was the only study to present a significance for outcomes measuring hip function (HHS and modified HHS) in THA patients, which favoured the outpatient group at a two year follow up of participants [57]. No study investigating a TKA population showed a difference in functional outcomes assessed between the two groups.

#### Satisfaction and quality of recovery

Across the included studies there was no significant difference between outpatient and inpatient groups for patient-reported satisfaction in both the THA and TKA populations assessed. Postoperative day one Quality of Recovery scores (QoR-9) presented favourably towards the outpatient group in one TKA study; however, each time point following indicated no significant difference in scores between groups [53]. There were no other outcome measures utilised in the THA or TKA studies that reported a statistical significance between the outpatient and inpatient groups.

## Discussion

Perioperative surgical and anaesthetic advancements, increased economic pressures, and the recognised need to maximise patient satisfaction after lower limb arthroplasty, have led to a rise in the number of THA and TKA procedures being performed in an outpatient setting. To the best of our knowledge, this is the first systematic review to explore the evidence for the effects of undergoing knee or hip arthroplasty in either an inpatient or outpatient setting on patient functional outcomes, the results of which demonstrated mixed support for either pathway.

A notable observation was that the majority of studies only utilised PROMs in their assessment and did not measure changes in physical performance outcomes such as gait, strength, endurance, or range of movement parameters. Additionally, the clinical and methodological heterogeneity across studies was considerable, multiple different functional outcomes were utilised and the assessment time points and follow-up periods of these also varied. The lack of randomisation in six out of the seven included trials is likely responsible for selection bias and the diversity of eligibility criteria within the included studies further limits the ability to compare the reported outcomes between groups. For these reasons, a meta-analysis was not feasible. However, despite the described limitations, this review still conjures some support for the outpatient setting as a good option for appropriately selected patients when other benefits of the pathway are considered.

Results for pain intensity across the studies were mixed. Some studies demonstrated a short-term (< 3 months) benefit for reduced pain following an inpatient pathway, however, the differences in pain decreased over time between both settings. The lower reporting of pain in the inpatient group in both THA and TKA study populations could potentially be explained by the outpatient group’s earlier mobilisation and more limited access to analgesia in the domestic environment [59]. In contrast, two THA studies found lower VAS scores in favour of the outpatient groups at short- and long-term assessment points [57, 58], these conflicting results are in line with existing evidence, demonstrating variation in pain scores between the two settings [24, 42].

Interestingly, functional outcomes in one study investigating a THR population (HHS and mHHS) favoured the outpatient pathway at a follow-up of two years, which cannot be explained by between-group differences given the inpatient and outpatient populations were case-matched [57]. Another study, this time investigating a TKA population, reported on improved quality of recovery (QoR-9) for the outpatient group compared to the inpatient group on post-operative day one [53]. This is an important finding as the inpatient setting provides greater access to resources in the acute post-operative period than those who are discharged the same day; however, this did not appear to be a significant factor in patient reporting of their quality of recovery from the TKA procedure.

The clinical implications of these results provide evidence suggesting non-inferior functional outcomes for an outpatient pathway when compared to a traditional inpatient stay after THA or TKA, which further strengthens the support for this option in selected patients. For outcomes assessing pain, self-reported function and quality of life there were no results which solely favoured the inpatient group in the included studies. Additionally, the lack of significance in difference across all other functional outcomes assessed, suggests that outpatient lower limb arthroplasty does not result in poorer self-reported outcomes or outcomes of physical performance when compared to an inpatient setting.

The observations of this review can assist to guide future research comparing inpatient to outpatient settings for TKA or THA. With respect to outcome measures, validated assessments of physical function should be included rather than relying on PROMs alone. As although PROMs capture a person’s perception of their own health and physical function, they lack the objectivity that performance-based physical assessments provide. Further, of the 55 studies assessed for eligibility at full-text, 35 were excluded for not including a functional outcome of any type. An additional recommendation based on the results of this review would be to clearly define and report eligibility criteria for each pathway, and where possible, consider randomisation to mitigate the effects of selection bias within trials.

## Conclusion

The results of this systematic review suggest that outpatient or inpatient pathway selection for hip or knee arthroplasty should not be based on the superiority of functional outcomes alone. However, given there is growing evidence in support of an outpatient pathway in select patients with respect to cost savings and without any increase in complications, it could be proposed that an equivalency of post-operative function between the two settings makes same-day discharge favourable.

### Supplementary Information

**Additional file 1.** PRISMA 2020 Checklist.

## Data Availability

Not applicable.

## Appendix 1

See Table 9.

**Table 9:** Database search strategies

Database	Search strategy
PubMed	(("outpatient"[Title/Abstract] OR "ambulatory surg*"[Title/Abstract] OR "day of surgery"[Title/Abstract] OR "same day discharge"[Title/Abstract])) AND (("knee replacement"[Title/Abstract] OR "knee arthroplasty"[Title/Abstract] OR "hip replacement"[Title/Abstract] OR "hip arthroplasty"[Title/Abstract] OR "lower limb arthroplasty"[Title/Abstract]))
CINAHL	(((TI outpatient OR AB outpatient) OR (TI "ambulatory surg*" OR AB "ambulatory surg*") OR (TI "day of surgery" OR AB "day of surgery") OR (TI "same day discharge" OR AB "same day discharge"))) AND (((TI "knee replacement" OR AB "knee replacement") OR (TI "knee arthroplasty" OR AB "knee arthroplasty") OR (TI "hip replacement" OR AB "hip replacement") OR (TI "hip arthroplasty" OR AB "hip arthroplasty") OR (TI "lower limb arthroplasty" OR AB "lower limb arthroplasty")))
EMBASE	((outpatient:ti,ab OR "ambulatory surg*":ti,ab OR "day of surgery":ti,ab OR "same day discharge":ti,ab)) AND (("knee replacement":ti,ab OR "knee arthroplasty":ti,ab OR "hip replacement":ti,ab OR "hip arthroplasty":ti,ab OR "lower limb arthroplasty":ti,ab))

## Appendix 2

See Table 10.

**Table 10:** JBI critical appraisal score

	Q1	Q2	Q3	Q4	Q5	Q6	Q7	Q8	Q9	Q10	Q11	Raw score/11	(%)	Quality Rating
JBI critical appraisal checklist for *cohort studies*														
Gabor, 2020	N	Y	Y	Y	Y	Y	Y	Y	U	U	Y	8	72.7	Good
Gauthier-Kwan, 2018	Y	Y	Y	Y	Y	Y	Y	Y	Y	U	Y	10	90.9	Good
Husted, 2020	Y	Y	Y	Y	Y	Y	Y	Y	U	U	Y	9	81.8	Good
Kolisek, 2009	N	Y	Y	Y	U	Y	Y	Y	Y	Y	U	8	72.7	Good
Zomar, 2020	U	Y	Y	Y	Y	Y	Y	Y	U	U	Y	8	72.7	Good

	Q1	Q2	Q3	Q4	Q5	Q6	Q7	Q8	Q9	Q10	Q11	Q12	Q13	Raw score/13	(%)	Quality rating
JBI critical appraisal checklist for *randomized controlled trials*																
Goyal, 2017	Y	Y	Y	N	U	U	Y	Y	Y	U	Y	Y	Y	9	69.2	Good

	Q1	Q2	Q3	Q4	Q5	Q6	Q7	Q8	Q9	Q10	Raw score/10	(%)	Quality rating
JBI critical appraisal checklist for *case–control studies*													
Rosinsky, 2020	Y	Y	Y	Y	Y	Y	Y	Y	Y	Y	9	90.0	Good

*GMQ*—good methodological quality, *N*—did not meet criteria, *U*—unclear, *Y*—met criteria

## Abbreviations

ADL: Activities of daily living
ASA: American Society of Anaesthesiologists Physical Status Classification system
BMI: Body mass index
DOS: Day of surgery
F: Female
HHS: Harris Hip Score
IP: Inpatient
JBI: Johanna Briggs Institute
M: Male
NHMRC: National Health and Medical Research Council
NR: Not reported
nSDD: Not same-day discharge
OA: Osteoarthritis
OARA: Outpatient arthroplasty risk assessment
OP: Outpatient
POD: Post-operative day
PRISMA: Preferred reporting items for systematic reviews and meta-analyses
PROMs: Patient-reported outcome measures
QoR: Quality of recovery
SD: Standard deviation
SDD: Same-day discharge
SR: Sport and recreation
THA: Total hip arthroplasty
TKA: Total knee arthroplasty
TUG: Timed up and go
UKA: Unicompartmental knee arthroplasty
VAS: Visual Analogue Scale
